# Evaluation of a New Patellar Tendon Bearing Brace With Offloading Monitoring and Adjustability: A Pilot Study

**DOI:** 10.1002/hsr2.70244

**Published:** 2024-12-17

**Authors:** Vahid Chamani, Mahmood Bahramizadeh, Mobina Khosravi, Akbar Biglarian, Gholamreza Ghorbani Amjad, Seyyed Mohammad Ebrahim Mousavi, Mokhtar Arazpour

**Affiliations:** ^1^ Department of Orthotics and Prosthetics University of Social Welfare and Rehabilitation Sciences Tehran Iran; ^2^ Physiotherapy Research Center, Department of Orthotics and Prosthetics, School of Rehabilitation Shahid Beheshti University of Medical Sciences Tehran Iran; ^3^ Department of Biostatistics and Epidemiology, Social Departments of Health Research Institute University of Social Welfare and Rehabilitation Sciences Tehran Iran; ^4^ Department of Orthopedics, School of Medicine Hamadan University of Medical Sciences Hamadan Iran

**Keywords:** adjustability, offloading, patellar tendon‐bearing brace

## Abstract

**Background and Aims:**

The patellar tendon‐bearing (PTB) brace is a crucial device designed to lessen axial forces on the tibia. The newly designed PTB brace allows clinicians to measure offloading amount in the realtime. This study aimed to explore the relationship between a rise in displacement between the foot plate and calf shells of this new PTB brace and changes in the amount of offloading on the tibia.

**Methods:**

This pilot study used a sample of five individuals with midshaft tibia fractures to investigate the effectiveness of the PTB brace under different conditions. The PTB brace was tested in six different conditions, with the displacement between the foot plate and calf shells varying in increments of 0.5 cm, from 1 cm to 3.5 cm.

**Results:**

The new PTB brace provided varying levels of offloading, ranging from 22% to 38%. As the vertical distance between the calf shells and foot plate increased, the offloading levels also increased significantly. The study observed significant differences in weight reduction (*p* = 0.02) and offloading percentages (*p* = 0.048) when comparing 1 cm and 1.5 cm displacement.

**Conclusion:**

The findings suggest that maintaining a distance of 1.5 cm or more between the calf shells and foot plate is effective in reducing weight and offloading on the tibia. These results have important implications for clinicians using PTB braces to treat tibia fractures, highlighting the importance of adjusting the displacement of the PTB brace to optimize patient outcomes.

## 1 Introduction

The patellar tendon‐bearing (PTB) brace was introduced by Dr. Sarmiento in the 1960s as a treatment option for tibia fractures [1]. This innovative device works by increasing the space between the calf clamp shells and the foot plate, reducing pressure on the tibia, fibula, and foot bones. The lateral metal uprights provide stability and control, promoting healing at the fracture site [2]. The PTB brace has become a popular alternative to traditional PTB casts due to its ease of cleaning, convenience, and improved hygiene [3].

However, despite its widespread use, the PTB brace has several limitations. One of the most significant challenges is the lack of quantification of the amount of offloading required for optimal healing. Current PTB braces do not provide clinicians with a reliable method to measure the amount of offloading, which can lead to suboptimal treatment outcomes. This lack of quantification can result in inadequate or excessive pressure on the tibia, fibula, and foot bones, potentially hindering the healing process.

According to previous studies, the orthotist provides offloading by increasing the flexion angle of the leg [4] and the longitudinal distance of the leg and foot plate [5, 6]. Also, a review of limited articles available in this field indicated that plantar pressure measurement is done by Pedar and scan devices [7], which is an effective research method for detecting the amount of offloading in the PTB brace [4, 7, 8]. Because of the high cost of these instruments for measuring plantar pressure, it is impossible to benefit from them in all clinics; they are used only in well‐equipped research centers and for research studies; thus, it is not cost‐effective to use them at the clinical level.

Our recent study aimed to address this limitation by designing and manufacturing a new PTB brace with built‐in monitoring system. The new PTB brace allows clinicians to measure and quantify offloading in realtime, providing a valuable tool for monitoring and adjusting offloading levels based on the specific needs of each patient and the stage of fracture healing [9].

In this study, we aim to explore the relationship between a rise in displacement between the foot plate and calf shells of this newly designed PTB brace and changes in the amount of weight reduction and offloading in the tibia. We investigated how this relationship changes, and how it can be used to inform treatment decisions and improve patient outcomes.

## 2 Materials and Methods

### 2.1 Participants

Five patients with midshaft tibia fractures, which were selected based on their clinical presentation and medical history were enrolled in the current pilot study. All patients signed a written informed consent form before enrollment. Participants with no neuromuscular diseases, no history of surgery in the lower limbs, no wounds or skin diseases and allergies, and no use of walking aids were included in this study. On the other hand, those having a difference in the length of the lower limbs and suffering from any physical and mental disabilities were excluded from the investigation. This study was approved by the Ethics Committee of the Rehabilitation and Social Welfare University (IR. USWR. REC.1400.266). All patients signed a written informed consent form before enrollment.

### 2.2 Novel PTB Brace

The design process of the novel PTB brace involved a thorough understanding of the biomechanical principles involved. The fabrication process consisted of three parts: design and fabrication of metal bars and ankle joints, design and fabrication of custom‐made bivalve calf shells and foot plates, and the electric part.

The materials used in the fabrication process were chosen for their durability, flexibility, and resistance to corrosion. The metal uprights, for example, were made of stainless steel 430, which was chosen for its ability to withstand frequent use and cleaning. The rationale for selecting this material was based on its ability to provide a comfortable and secure fit for the patient.

The load cells used in the PTB brace were Kelly brand DEE C3, which were chosen for their high accuracy and reliability. The characteristics of the load cells were carefully considered, including their capacity, accuracy class, material, sealing degree, and effect of temperature on sensitivity. The load cells were connected to the side uprights of the brace and their output was connected to the processor system, which showed the maximum and minimum vertical loads on the side uprights.

The design and fabrication of the custom‐made bivalve calf shells and foot plates involved a careful consideration of the volume of the limb and the need to prevent limb pistoling within the PTB brace. The shells were designed to accommodate the limb volume and were fabricated using 4 mm polypropylene sheets. The foot plate was also constructed using a 5‐mm polypropylene sheet.

**FIGURE 1:**
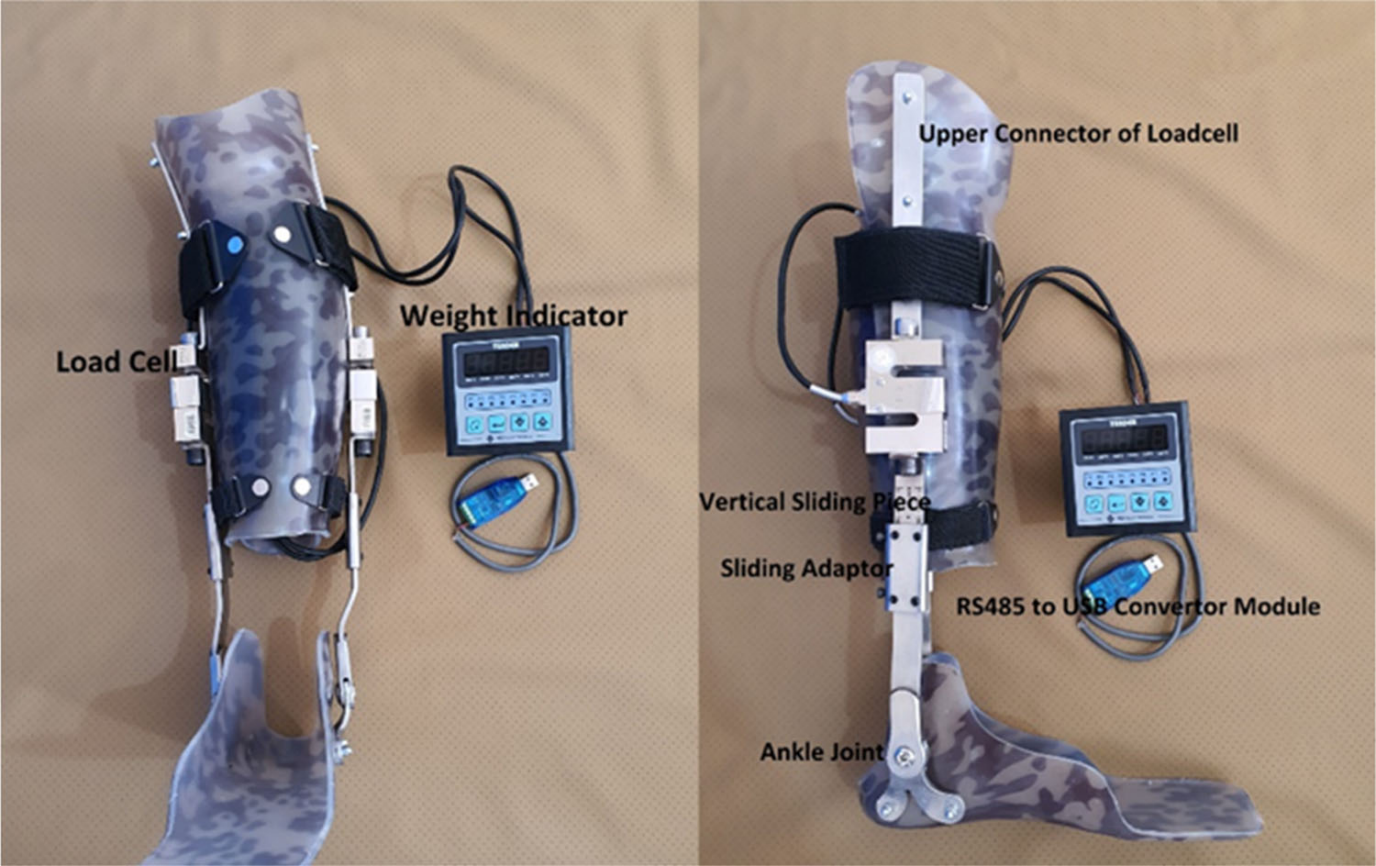
Weight indicator PTB orthosis.

The electric part of the PTB brace consisted of a weight indicator (Trademark of Trancel, Model: Tender WI610) with an RS485 output cable, which was used to receive load cell pulsations and indicate the amount of load being carried by the PTB brace. The Tender WI610 has a sensitivity of 1 mV/V, allowing it to detect changes in load with a resolution of 1 mV. Its accuracy is ±0.5% of the full scale range (FSR), which is 600 kg (1323 lbs) for this model, ensuring that the weight indicator can measure the weight being carried by the PTB brace with an accuracy of ±3 kg (±6.6 lbs). Additionally, the Tender WI610 has a linearity error of ±0.5% of FSR and a noise resistance of 100 dB, ensuring that its output is linearly proportional to the applied load and can withstand electrical noise and vibrations that may be present in the environment. The weight indicator was calibrated by configuring the load cells, as shown in Figure 1.

All data about this procedure are described in our recent article [9].

### 2.3 Measurement Tool

**FIGURE 2:**
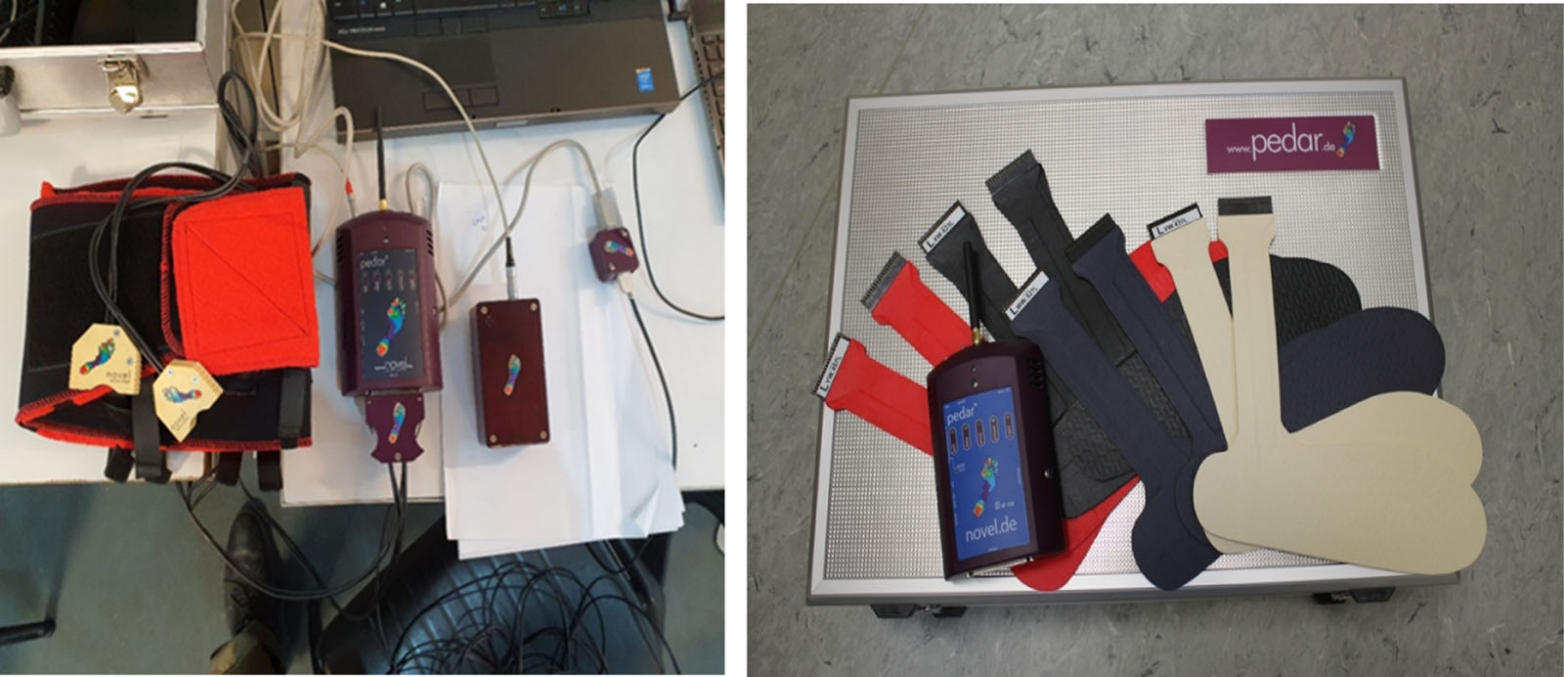
Novel Pedar‐X in‐shoe pressure measuring system (Novel GmbH, Munich, Germany).

A novel Pedar‐X in‐shoe pressure measuring system (Novel GmbH, Munich, Germany) was used to measure plantar pressure in the foot area. The repeatability of this device was demonstrated in a study by Ramanathan et al. [10]. The foot‐shaped sensors were customized to fit each subject's foot size and placed between the plantar surface of the foot and the foot plate of the PTB brace (Figure 2).

### 2.4 Data Collection

The PTB brace was used in this study based on the validated and reliable design established in our previous study [9]. The PTB brace was custom‐made for all five patients who were included in the study. Six conditions of offloading levels were applied to the tibia by changing the distance between the shells and the foot plate. This distance was 1, 1.5, 2, 2.5, 3, and 3.5 cm. The offloading percentages of the brace were changed by increasing the distance.

The subjects walked at a self‐selected speed for almost 5‐min to get used to the settings. After a 5‐min rest, each subject walked on an 8‐m straight pathway. Each condition was tested three times. The values displayed during each offloading condition while walking were recorded as a percentage on the load cell display. The maximum values recorded during each test were captured and saved in an Excel file at the end of each test using Trancel software. After accomplishing each condition, the brace was removed from the participant's foot, and a wash‐out time of at least 10‐min between different test conditions was considered [11]. Different distances were randomly adjusted to prevent any inadvertent potential effect of the gradual increase in the load level.

### 2.5 Statistical Analysis

SPSS software 26 (IBM Corp, and Armonk, New York, USA) was used for data analysis. The Shapiro–Wilk test was used to check data normality. A repeated‐measures analysis of variance was conducted to determine the differences between the six experimental conditions, and Tukey's multiple comparison test was performed as the post hoc test to determine the differences between each condition. The significance level was considered 0.05.

## 3 Results

Five patients with an average age of 45.8 (±13.737) years, height of 1.63 m (±5.14), and body mass index of 27.32 kg/m^2^ (±2.6574), and average weight of 69.58 kg (±10.296) completed six test conditions.

The means and standard deviations (SD) for weight measured by PTB brace load cells and percentages of reduction in each condition are listed in Table 1.

**TABLE 1:** The mean (standard deviation) and percentages of weight shown in load cells while walking.

Variable	1 cm	1.5 cm	2 cm	2.5 cm	3 cm	3.5 cm
Weight reduction	16.06 (7.05)	23.86 (10.25)	24.26 (9.95)	24.76 (12.14)	25.34 (12.22)	28.30 (11.36)
	21.92%	32.54%	33%	33.68%	34.48%	38.32%

Table 2 compares the weight reduction and offloading percentages of the PTB brace under different offloading conditions.

**TABLE 2:** Results of the comparison between six test conditions.

Test conditions (cm)	Weight reduction	Offloading percentages
1		
1.5	0.020*	0.048*
2	0.432	0.338
2.5	0.630	0.512
3	0.614	0.698
3.5	0.220	0.217
1.5		
2	1.00	1.00
2.5	1.00	1.00
3	1.00	1.00
3.5	1.00	1.00
2		
2.5	1.00	1.00
3	1.00	1.00
3.5	0.759	1.00
2.5		
3	1.00	1.00
3.5	0.613	1.00
3		
3.5	1.00	1.00

* indicates significant level.

Based on the results (Table 1), an increase in the vertical distance between the calf shells and foot plate of the PTB brace led to an increase in offloading. The average minimum offloading in the 1‐cm sliding adapter position was approximately 22%, whereas the maximum average offloading in the 3.5‐cm sliding adapter position was 38%. The percentage difference on average offloading from 1 to 1.5 cm, 1.5 to 2 cm, 2 to 2.5 cm, 2.5 to 3 cm, and 3 to 3.5 cm was 10.62%, 0.46%, 0.68%, 0.8%, and 3.84%, respectively.

As a result, the weight reduction (*p* = 0.020) and offloading percentage (*p* = 0.048) significantly differed when comparing the displacement of 1 cm with that of 1.5 cm. However, no significant differences were observed between the other conditions, indicating that the amount of offloading does not change significantly more than 1.5 cm displacement between the foot plate and calf shells of the PTB brace.

## 4 Discussion

This study aims to investigate the correlation between the increase in displacement between the foot plate and calf shells of the newly designed PTB brace and changes in the amount of weight reduction in the tibia. Our findings indicated that 1.5 cm or more between the foot plate and calf shells can significantly decrease the weight on the tibia.

The findings demonstrated that by increasing the vertical distance between the calf shells and foot plate of the PTB brace, the weight reduction was linear. In none of the various conditions did the PTB brace represent a 100% offloading level (Whether with a Pedar or PTB brace load cells). This could be due to the effect of calf muscle activity on the midstance and preswing phases of walking. Regarding the evaluation of the performance of the PTB brace on patients, similar to the results obtained from the study of offloading levels in healthy individuals, none of the patients achieved 100% offloading, with the mean maximum being 38%, which can again be attributed to the effect of calf muscle activity.

The percentage difference on average offloading from 1 to 1.5 cm was 10.62%, which was significant. Based on these results, the maximum and minimum difference in offloading occurred from 1 to 1.5 cm and from 2 to 2.5 cm displacement, respectively. Therefore, there will be no significant changes in offloading beyond 1.5 cm, likely due to increased movement and slipping of the leg on the calf shells within the range of 2–3.5 cm. Another strong possibility is that the increased displacement of fracture fragments causes more pain, leading the patient to bear less weight on the affected limb. Consequently, offloading does not undergo a meaningful change from the range of 1.5 cm onwards. Currently, the recommended offloading height used in clinics is 1 cm [4], and clinicians are advised to use the tables derived from this study specifically for patients with tibia bone fractures while increasing the offloading to 1.5 cm for optimal offloading in patients with midshaft tibia fractures.

In the design of this brace, foam padding was used to increase the friction between the body and the brace. It is suggested to use silicone padding instead to maximize this friction and prevent excessive movement of the body part inside the brace. The new PTB brace is studied only for patients with midshaft fractures of the tibia bone, and it should be investigated in cases with more proximal fractures of the tibia shaft. One of the limitations of our pilot study was the small sample size, which may have limited our ability to generalize our findings. Future studies should aim to recruit a larger sample size with long‐term effects. Additionally suggest investigating the effects of varying levels of physical activity on the weight indicator's accuracy and sensitivity, evaluating the performance of the weight indicator during different phases of PTB brace wear, such as initial wear and long‐term wear, and assessing the impact of environmental factors, such as temperature and humidity, on the weight indicator's accuracy and sensitivity. Also, our study did not thoroughly evaluate the user interface of the offloading mechanism, which could impact its usability for patients. We also did not assess the potential risks of improper adjustment or incorporate patient feedback and preferences into our design. To improve future studies, we recommend conducting user testing and gathering patient feedback to identify areas for improvement. Additionally, we suggest incorporating patient feedback and preferences into the design of the offloading mechanism to allow patients to adjust the device based on their individual needs and comfort levels.

## 5 Conclusion

The findings suggest that the PTB braces with a load cell monitoring system can significantly impact the weight reduction of the tibia bone when the distance between the calf shells and foot plate is greater than 1.5 cm. This monitoring system enables clinicians to accurately measure offloading and adjust treatment accordingly, taking into account individual patient needs and fracture healing stages. As a result, this innovative brace has the potential to improve fracture treatment outcomes and expedite patients' recovery, ultimately enabling them to return to their daily activities sooner.

## Author Contributions

**Vahid Chamani:** project administration, conceptualization, investigation, data curation. **Mahmood Bahramizadeh:** investigation, resources, supervision, methodology, project administration. **Mobina Khosravi:** writing–original draft, writing–review and editing, investigation, formal analysis. **Akbar Biglarian:** formal analysis. **Gholamreza Ghorbani Amjad:** data curation. **Seyyed Mohammad Ebrahim Mousavi:** conceptualization, project administration. **Mokhtar Arazpour:** supervision, methodology, project administration.

## Conflicts of Interest

The authors declare no conflicts of interest.

## Transparency Statement

The lead author Mokhtar Arazpour affirms that this manuscript is an honest, accurate, and transparent account of the study being reported; that no important aspects of the study have been omitted; and that any discrepancies from the study as planned (and, if relevant, registered) have been explained.

## Data Availability

The data that support the findings of this study are available from the corresponding author upon reasonable request.
